# Health Literacy among Health and Social Care University Students

**DOI:** 10.3390/ijerph17072273

**Published:** 2020-03-27

**Authors:** Dolors Juvinyà-Canal, Rosa Suñer-Soler, Adela Boixadós Porquet, Marion Vernay, Hervé Blanchard, Carme Bertran-Noguer

**Affiliations:** 1 Health and Health Care Research Group, Department of Nursing, Faculty of Nursing, University of Girona, 17003 Girona, Spain; dolors.juvinya@udg.edu (D.J.-C.); carme.bertran@udg.edu (C.B.-N.); 2 Training and Research Unit, Social Work School, University of Barcelona, 08007 Barcelona, Spain; boixados.adela@gmail.com; 3 Cross-Border and European Projects Coordinator, Regional Institute of Social Work, 66000 Perpignan, France; marion.vernay@irtsperpignan.fr; 4 Laboratory Center for Economic and Development Law, University of Perpignan, 66000 Via Domitia, France; blanchard@univ-perp.fr

**Keywords:** health literacy, HLS-EU-Q16 questionnaire, health promotion, health literacy/classification, students/education

## Abstract

Health literacy has been defined by the World Health Organization as the cognitive and social skills which determine the motivation and ability of individuals to gain access to, understand and use information in ways which promote and maintain good health. Its importance in reducing inequalities makes health literacy a thematic area that should be addressed in the training of professionals in the fields of healthcare, Social Work and Education. The objective of this study was to define the health literacy levels of students from the Universities of Girona and Barcelona (Spain) and the Regional Institute of Social Work in Perpignan (France). A cross-sectional study was conducted among students of Nursing, Social Work, Primary Education and Special Education in the 2017–2018 academic year. Sociodemographic and academic variables were considered and the HLS-EU-Q16 questionnaire was used to study health literacy levels. In total, 219 students with an average age of 24.9 participated. Of these, 64.4% were studying Social Work, 23.7% Nursing, 5.9% Primary Education, and 5.9% Special Education. Of the total sample, 36.5% were classified as sufficient in health literacy. The total average score of the health literacy index was 11.1; 13.2 among Nursing students; 10.5 among Social Work students; 10.1 among Primary Education students, and 10.1 among Special Education students (*p* < 0.001). Nursing students obtained the best results and healthcare was the highest rated subdomain, more than disease prevention and health promotion.

## 1. Introduction

### 1.1. Health Literacy

Health literacy has been defined by the World Health Organization as the cognitive and social skills which determine the motivation and ability of individuals to gain access to, understand and use information in ways which promote and maintain good health [1]. In 2012, Sørensen et al., referred to the multidimensional nature of health literacy and defined it as a concept closely linked to literacy and entails people’s knowledge, motivation and competences to access, understand, appraise, and apply health information in order to make judgments and take decisions in everyday life concerning healthcare, disease prevention and health promotion to maintain or improve quality of life during the life course [2]. Moreover, healthcare professionals and health systems play an essential role in facilitating this process and therefore personnel training is essential.

### 1.2. Health Literacy Levels

The European Health Literacy Survey revealed that almost half of the people (47.6%) surveyed in Europe had limited (insufficient or problematic) health literacy, and in Spain this percentage was higher (58.3%) [2]. However, the percentage of the population with an inadequate level of health literacy in Catalonia (northeast Spain) was 10.3% and 5.1% with a problematic level of health literacy [3]. In Asia, the level of health literacy among 224 students from two faculties (Science and Health Sciences) was measured. Study findings indicated that 93% of the students had insufficient health literacy [4]. Another study carried out in Turkey, among Nursing students, concluded that 29.3% of the students had an insufficient level of health literacy [5].

### 1.3. Research Justification

Improving health literacy is necessary to reduce health inequalities. For this purpose, good, reliable, accessible information, addressed to the needs and circumstances of different social groups, is required [6]. A direct correlation has been detected between low levels of health literacy and poor health, with a greater risk of hospitalization and mortality, especially among the elderly [7,8,9,10]. This makes health literacy a crucially important subject area that should be addressed in the training of professionals in the fields of health and social care and education. Including health literacy contents ensures that future healthcare professionals are adequately prepared to provide care, making it easier for people to understand information about healthcare [11], disease prevention and health promotion. Training health sciences students to assess legibility and editing in simple language can reduce patient literacy demands and meet the need for professionals with these skills [12]. Recent studies have endorsed the integration of health literacy in the curriculum for Nursing students [5]. A qualitative study carried out among healthcare professionals in the United Kingdom concluded that these professionals lacked both the confidence and the strategies to address the health literacy needs of their patients [13]. Nonetheless, including health education in training programs does not guarantee that students are health literate [14].

In a study exploring the health literacy profiles of Health Science students in Nepal, Budhathokia et al., found that students had moderate support and skills to manage their health, which was considered important for them. They also had a moderate ability to engage with healthcare professionals and the health system, highlighting the need for universities to take actions aimed at enhancing student access to health information throughout their training, and at developing the capacity of students to be actively involved with healthcare providers. In this way, society will have healthcare professionals with enhanced health literacy levels, sensitive to the health literacy needs of the different population groups they will have to care for once they embark on their professional careers [15].

Another study conducted by Rababah et al., among college students in northern Jordan showed that the level of health literacy among college students was influenced by demographic characteristics and that the success of health promotion lay in highlighting the key role played by disease prevention and health improvement in populations with less chronic health problems, such as the student population. The authors emphasized the usefulness of inter-professional education in optimizing the health outcomes of college students [16].

Health, Education and Social Work professionals play a leading role in promoting health literacy among citizens. Consequently, it is important they receive a good preparation in this field. Therefore, the aim of this research was to study the levels of health literacy among students studying degrees in Nursing, Social Work, and Primary Education.

## 2. Materials and Methods

### 2.1. Study Design and Participants

A cross-sectional study was carried out by the University of Girona and the University of Barcelona in Catalonia (Spain), and the Regional Institute of Social Work in Perpignan (France), in the framework of the European Union PROSPECTSASO Project. Participants were final-year students in the degrees of Nursing, Social Work, Primary and Special Education. In the case of the Spanish students their final year was their fourth, whereas in the case of the French students it was their third. Before the end of the 2017–2018 academic year, students were invited to participate in the study at the start of a teaching session accompanied by a teacher or research collaborator.

### 2.2. Measurement Instruments

Sociodemographic variables (age and sex), academic characteristics (type of ongoing studies, previous studies, work activity, university and country) were studied using an ad-hoc questionnaire. Health literacy levels were analysed using the HLS-EU-Q16 questionnaire, which consists of 16 questions that classify the degree of perceived difficulty (very easy, easy, difficult, very difficult, or do not know/does not answer) in each situation proposed in the survey. When a participant did not respond to at least 14 of the 16 questions, he or she was excluded from consideration. Two students were excluded for this reason, both having failed to respond to four questions. Items related to health care (7 out of 16 questions, disease prevention (5 out of 16) and health promotion (4 out of 16). Responses were dichotomised, with “very easy” and “easy” given a score of 1, and “difficult” and “very difficult” given a score of 0. Each participant’s score was obtained by adding up the scores for each item. To calculate the HLS-EU-Q16 index, scores were ranked: (0–8) Inadequate, (9–12) Problematic, and (13–16) Sufficient [17].

The HLS-EU-Q16 pointed out the high reliability (Cronbach’s alpha: 0.982) of the psychometric properties of this questionnaire in a recent Spanish sample made up of 5,485 individuals aged 15 or over [18]. The students were administered the validated questionnaires for their respective languages [18,19]. The reliability of the questionnaire in this present sample (Cronbach’s alpha: 0.864) was also high.

### 2.3. Ethical Considerations

The study was approved by the management committee at each academic centre. Participation was voluntary and anonymous. This project respects the Helsinki Declaration of the World Medical Association on the ethical principles for medical research involving human subjects and the ethical principles with regards to research set out in Organic Law 3/2018 regarding Personal Data Protection.

### 2.4. Statistical Analysis

The IBM SPSS Statistics^®^ V25 software package (Madrid, Spain) was used for data analysis. Mean and standard deviation were used to describe the quantitative variables, and absolute frequency and its percentage were used to describe the categorical variables.

The Student’s *t*-test was used to study the relationship between variables that met the criteria for normality. ANOVA was used to compare quantitative with categorical variables, and the Chi-Square test was used for the categorical variables. In addition, multiple binary regression was carried out to study variables associated with health literacy, distinguishing levels for this index as sufficient or problematic (between 0 and 12 points) and sufficient (between 13 and 16 points). The level of statistical significance was considered to be *p* < 0.05.

## 3. Results

### 3.1. Characteristics of the Study Population

Two hundred and nineteen students (187 women (85.4%) and 32 men 14.6%) took part in the study. The average age was 24.9 years (SD 5.2) (the youngest was 19 and the oldest 60), with no differences according to sex (women’s average age was 24.8; men’s average age was 25.3; *p* = 0.629).

The majority of students (64.4%) were studying a degree in Social Work (n: 141); 23.7% (n: 52) were studying a degree in Nursing; 5.9% (n: 13) a degree in Primary Education, and also 5.9% (n: 13) a degree in Special Education. In addition, 78.2% of the participants combined work and studies and 4.1% (n: 9) reported having another degree.

### 3.2. Health Literacy Results

Table 1 shows the scores for each item on the HLS-EU-Q16 questionnaire based on the type of degree participants were studying, with descriptions obtained from the original scores (very difficult = 1, difficult = 2, easy = 3 and very easy = 4).

The highest mean scores corresponded in most of the 16 items to Nursing students with significant differences, except in item 2 (—find out where to get professional help when you are ill?), 10 (—understand why you need health screenings?), and 15 (—understand information in the media on how to get healthier?), in which the scores obtained by students of the four degrees were not statistically significant.

Table 2 shows the average scores by subdomains of the HLS-EU-Q16 questionnaire. In the total sample, the highest scores corresponded to the healthcare subdomain. By type of studies, Nursing students had significantly higher scores in the three subdomains (*p* < 0.01).

The total average score obtained from the participants’ health literacy index was 11. 1 (± 3.2) using the HLS-EU-Q16 questionnaire, after recoding answers and transforming each item into a dichotomous response (difficult and very difficult = 0; easy and very easy = 1).

By gender, the average health literacy index score was 11.1 (±3.1) for women and 10.8 (±3.6) for men (Student’s *t*-test; *p* = 0.593). By university degree, the average health literacy index score for Nursing student participants was 13.2 (± 4); 10.5 (± 2.9) for Social Work students; 10.1 (± 2.8) for Primary Education students, and 10.1 (± 2.7) for Special Education students (ANOVA analysis; *p* < 0.001).

Participants who reported having another degree presented higher average health literacy index scores (12.3 versus 11.1; (Student’s *t*-test; *p* = 0.255).

**Figure 1:**
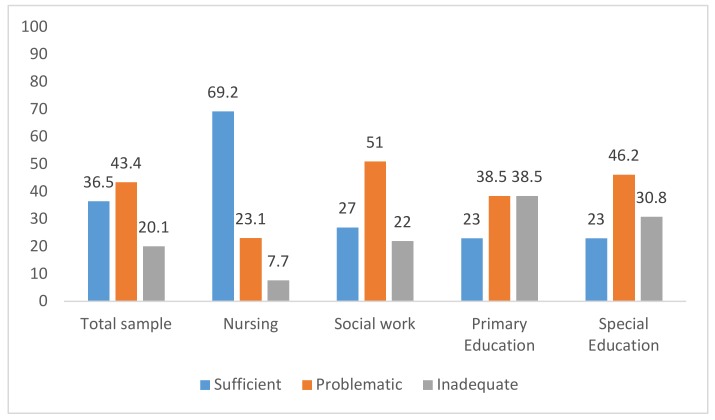
Levels of health literacy index by type of degree and for the total (%). HLS-EU-Q16 index: (score 0–8) Inadequate, (score 9–12) Problematic, and (score 13–16) Sufficient.

Figure 1 shows the classification of the participants’ levels of health literacy (insufficient, problematic or sufficient) in the total sample and by type of degree. Almost 7 out of 10 participants in the Nursing group and almost 3 out of 10 in the Social Work group obtained this level, while approximately a quarter of the Primary Education and Special Education students were in this category.

On studying the variables associated with health literacy levels using the binary multiple regression model adjusted for sex and age (Table 3), it was observed that the university degree variable was associated with health literacy. Thus, participants studying Social Work, Primary and Special Education were less likely to be classified at a sufficient level of health literacy than participants studying Nursing (Social Work OR 0.15 IC 95% 0.07–0.31; *p* < 0.001; Primary Education OR 0.10 IC 95% 0.02–0.46; *p* < 0.003; Special Education OR 0.05 IC 95% 0.00–0.31; *p* = 0.001). In addition, having another degree was also related to health literacy so participants with another university degree were more likely to have sufficient health literacy (OR 0.19 IC 95% 0.03–0.99; *p* = 0.049).

## 4. Discussion

In this study, we defined the health literacy levels of undergraduate students in the fields of health, Social Work and Education in two universities in Spain and in a Social Work training institute in France. Among the sociodemographic variables, it is worth noting the large number of women participating in the study, as well as the average age of 25 years, which was similar for both sexes. This can be accounted for by the type of degree and the highly feminized fields of health and education covered by this research.

The average health literacy score of the participants was 11.1 and they had very differentiated and significant results depending on the degree they were studying. Thus, the average level of literacy was clearly higher among Nursing students (13.2) and lower among Social Work students (10.5) and Primary and Special Education students (10.1). The fact that participants with another degree had higher health literacy levels (12.3) would suggest that their age was related, favouring greater contact with the health system and its professionals [2].

Women had slightly higher literacy scores than men, although not significantly. The related literature offers mixed evidence for the relationship between gender and health literacy of college students. In a study by Garcia-Codina et al., women showed slightly higher percentages of inadequate/problematic health literacy in a much larger sample with a tendency to statistical significance [3], the same was observed in the study by Rababah et al. [16] and in the study by Vamos et al., in Texas [20]. However, other authors have not found these differences [21].

The fact that Nursing and Medical students have better literacy outcomes has been found in several studies conducted in the university environment, such as the one carried out by Rababah et al., which also highlighted the influence of demographic characteristics, such as age, gender, field of study and academic year, on the literacy level of college students. Health Science students, women and non-smokers had better health literacy [16]. Other authors, such as Mather at al. in a study conducted among students in Tasmania, have identified the importance of these variables together with the parents’ level of education. They highlighted the literacy deficiencies of Health Science students and pointed out the need to incorporate health literacy in secondary and tertiary curricula in order to promote it among the general population and especially among future care providers [22].

It is worth highlighting that although one third of the participants were ranked sufficient in health literacy, the Nursing group had more students at this level (seven out of ten). This ratio decreased to more than half of the participants in the Social Work and Education groups. Thus, Nursing students obtained the highest average scores in all the aspects covered in the questionnaire except those related to finding out where to get professional help when you are ill, understanding why you need health screenings, and understanding information in the media on getting healthier, in which the scores obtained by students of the four degrees were not statistically significant. The fact that participants studying to become nurses obtained these results could be due to the learning objectives themselves and degree competencies and contents. However, it is necessary to take into account the importance of personal background (family and social environment, previous health-related experiences, etc.) and the students’ educational trajectories so a good approximation can be made to their health literacy levels. In this way, both specific and cross-curricular literacy-related training and curricular activities can be planned, as illustrated by Elsborg et al., in a study conducted among Danish students enrolled in health-related study programs [21].

Nursing students had similar results in limited (inadequate and problematic) health literacy to the general population of Spain [17], but not of Catalonia [3] and Valencia [18] (30.8% versus 34.4%, 15.6% and 12.8% respectively). Students of Special Education, Primary Education and Social Work had less favourable health literacy results than the reference populations. In addition, the number of students (over 70%) with very high limited literacy levels was striking. The results show the need for cross-cutting lines of action to be implemented for the enhancement and empowerment of university students in health-related matters. Studies such as the one by Ozen et al. [5] endorse the effectiveness of improving health literacy among Nursing students by including it in the education program. Bröder et al., stated that the more health literate students are, the greater the influence they will have on people in their professional lives in terms of health management and healthier decision-making [23]. Nurses need to be able to tailor care to people with low levels of health literacy, making it essential to integrate health literacy into Nursing studies. Authors such as Mosley and Taylor have assessed literacy needs and identified points for improvement in order to construct a health literacy plan integrated in the Nursing curriculum that includes learning activities based on evaluation and effective communication [24].

Nursing students reported having fewer difficulties carrying out healthcare, health promotion and disease prevention actions than students of Primary Education and Special Education and Social Work. However, training that goes well beyond knowledge of healthcare and caring for people with health problems is needed. Self-care competencies should be incorporated and reinforced in Nursing programs to enable Nursing students to transmit this knowledge in their future professional careers. This is also an essential requirement for graduates in education and community work. Ayaz-Alkaya and Terzi, have highlighted the importance of health literacy especially for Nursing students given that nurses are key players in addressing the problem of low health literacy by improving the health outcomes of the people they care for through health education and health promotion. Health education is a fundamental responsibility for nurses, and it is imperative they have sufficient health literacy skills [25].

In general, actions such as judging when you need to get a second opinion from your doctor, finding information on how to manage mental health problems and judging if the information on health risks in the media is reliable were considered the most difficult to perform. This may be related to the generational component.

This study reaffirms that gender is not related to health literacy levels. However, it contradicts the belief that people’s educational level is a protective factor given that the sample corresponded to university students and that 63.5% of these students had limited health literacy, which was well above the percentage in the reference populations. This result conflicts with the findings of the study on health literacy determinants conducted by Garcia et al., among the general population in Catalonia [3].

The average age of the sample in relation to the general populations of the reference studies in Spain and Catalonia may have influenced the lower levels of limited literacy, since younger people are less likely to have limited literacy levels, as pointed out in studies carried out by Sørensen et al. [2]. However, Bröder et al. [26] argued that young people have unique characteristics in terms of needs, assets and perspectives, and therefore should be considered a specific group regarding health literacy and its determinants. They proposed a six-dimensional model including, among other determinants, not only young people’s sociodemographic characteristics, but also their health perspectives and the digital world in which they have grown [26].

### 4.1. Limitations

As far as study limitations are concerned, the sample size, especially of the Primary and Special Education groups, may be biased due to lack of representation. Accordingly, the results should be treated with caution. In addition, the cross-sectional design does not allow for causal relationships to be established, since only some possible factors associated with literacy levels in a population of university students have been analysed. Nonetheless, despite the scant literature on health literacy among college students, we consider it of great interest to study health literacy in university students in order to work on preventing the potential effects that the low literacy levels observed may have on these people.

### 4.2. Practical Implications

The results of this study have shown the need to improve health literacy levels by incorporating additional cross-cutting actions in training programs aimed at health care, disease prevention and health promotion. Not only are these actions needed in Nursing studies but also in Social Work and Education degrees, given the importance of incorporating literacy in the early stages of education and through healthcare education and contact with the community.

## 5. Conclusions

Levels of health literacy among university students in the fields of health, social work and education were found to be limited, especially among students of Social Work, Infant and Special Education. Thus, Nursing students had better levels of health literacy.

Health care was the domain that obtained the best score among all the participants, more than disease prevention and health promotion. Aspects related to finding out where to get professional help when you are ill, understanding why you need health screenings and understanding information in the media on how to get healthier obtained the lowest scores among the participants of all the degrees studied. In addition, students with a previous degree had a better average level of health literacy.

## Figures and Tables

**Table 1:** Results of HLS-EU-Q16 scores in the total sample and by type of degree.

	Item HLS-EU-Q16	Total Sample N: 219	Nursing Students N: 52	Social Work Students N: 141	Primary Education Students N: 13	Special Education Students N:13	*p*
**1**	—find information on treatments or illnesses that concern you?	2.9 (0.6)	3.1 (0.5)	2.9 (0.6)	2.6 (0.8)	2.6 (0.7)	0.004
**2**	—find out where to get professional help when you are ill?	3.1 (0.6)	3.2 (0.6)	3.1 (0.6)	3 (0.9)	2.8 (0.5)	0.179
**3**	—understand what the doctor says to you?	2.8 (0.7)	3.2 (0.6)	2.7 (0.7)	3 (0.5)	3 (0.6)	0.000
**4**	—understand your doctor’s or pharmacist’s instructions on how to take a prescribed medicine?	3.2 (0.6)	3.4 (0.5)	3 (0.6)	3.5 (0.5)	3.1 (0.6)	0.002
**5**	—judge when you need to get a second opinion from your doctor?	2.4 (0.8)	2.7 (0.7)	2.3 (0.7)	2.2 (1)	2 (0.8)	0.002
**6**	—use information the doctor gives you to make decisions about your illness?	2.7 (0.7)	2.9 (0.6)	2.6 (0.7)	2.2 (0.6)	2.5 (0.4)	0.003
**7**	—follow instructions from your doctor or pharmacist?	3.1 (0.6)	3.4 (0.5)	3.1 (0.6)	3.1 (0.6)	3 (0.7)	0.031
**8**	—find information on how to manage mental health problems like stress or depression?	2.4 (0.8)	2.6 (0.8)	2.3 (0.8)	1.8 (0.6)	2.3 (0.7)	0.012
**9**	—understand health warnings about behaviour such as smoking, low physical activity and drinking too much?	3.2 (0.6)	3.4 (0.5)	3.1 (0.6)	3 (0.8)	3.3 (0.4)	0.013
**10**	—understand why you need health screenings?	3.1 (0.6)	3.2 (0.6)	3.1 (0.6)	3 (0.6)	3.4 (0.5)	0.123
**11**	—judge if the information on health risks in the media is reliable?	2.4 (0.7)	2.7 (0.5)	2.4 (0.7)	2 (0.7)	1.8 (0.5)	0.000
**12**	—decide how you can protect yourself from illness based on information in the media?	2.5 (0.7)	2.8 (0.6)	2.4 (0.7)	2.2 (0.7)	2.4 (0.7)	0.007
**13**	—find out what activities are good for your mental well-being?	2.6 (0.8)	2.7 (0.8)	2.4 (0.8)	3 (0.7)	2.9 (0.6)	0.022
**14**	—understand advice on health from family members or friends	2.9 (0.6)	3.2 (0.6)	2.8 (0.6)	3.2 (0.7)	2.6 (0.4)	0.000
**15**	—understand information in the media on how to get healthier?	2.8 (0.7)	3 (0.6)	2.8 (0.7)	2.6 (0.6)	2.6 (0.6)	0.062
**16**	—judge which everyday behaviour is related to your health?	2.9 (0.7)	3.2 (0.6)	2.8 (0.7)	2.8 (0.8)	2.6 (0.7)	0.002

Note: Test ANOVA. The descriptive variables (mean and standard deviation in brackets) are shown with the degree of perceived difficulty in each situation proposed in the survey (very difficult = 1, difficult = 2, easy = 3 and very easy = 4.).

**Table 2:** Average scores of the HLS-EU-Q16 subdomains in the total sample.

	Total Sample N: 219	Nursing Students N: 52	Social Work Students N: 141	Primary Education Students N:13	Special Education Students N:13	*p*
**Health care (HL-1, HL-2, HL-3, HL-4, HL-5, HL-6, HL-7)**	2.9 (0.4)	3.1 (0.4)	2.8 (0.4)	2.8 (0.4)	2.7 (0.3)	0.000
**Disease prevention (HL-8, HL-9, HL-10, HL-11, HL-12)**	2.7 (0.4)	3 (0.4)	2.7 (0.4)	2.4 (0.4)	2.6 (0.3)	0.000
**Health promotion (HL-13, HL-14, HL-15, HL-16)**	2.8 (0.5)	3 (0.5)	2.7 (0.5)	2.9 (0.4)	2.7 (0.3)	0.001

Note: Test ANOVA. The descriptive variables (mean and standard deviation in brackets) are shown with the degree of perceived difficulty in each situation proposed in the survey (very difficult = 1, difficult = 2, easy = 3 and very easy = 4.).

**Table 3:** Variables associated with sufficient health literacy using the binary multiple regression model.

	B	Standard Error	Wald	Sig.	OR	CI 95% OR	
						Lower	Higher
**Age**	**0.029**	**0.031**	**0.879**	**0.348**	1.030	0.968	1.095
**Sex (Male)**	0.283	0.430	0.435	0.509	1.328	0.572	3.081
**University Studies (Nursing)**			30.159	0.000			
**University Studies (Social Work)**	−1.858	0.361	26.494	0.000	0.156	0.077	0.316
**University Studies (Primary Education)**	−2.307	0.783	8.683	0.003	0.100	0.021	0.462
**University Studies (Special Education)**	−2.965	0.929	10.197	0.001	0.052	0.008	0.318
**Second Degree (No)**	−1.659	0.844	3.868	0.049	0.190	0.036	0.994
**Constant**	1.716	1.102	2.425	0.119	5.561		
